# Context-Aware Reviewer Assignment for Trust Enhanced Peer Review

**DOI:** 10.1371/journal.pone.0130493

**Published:** 2015-06-19

**Authors:** Lei Li, Yan Wang, Guanfeng Liu, Meng Wang, Xindong Wu

**Affiliations:** 1 School of Computer Science and Information Technology, Hefei University of Technology, Hefei, Anhui, China; 2 Department of Computing, Macquarie University, Sydney, Australia; 3 Soochow Advanced Data Analytics Lab, Soochow University, Suzhou, Jiangsu, China; 4 Department of Computer Science, University of Vermont, Burlington, Vermont, USA; Peking University, CHINA

## Abstract

Reviewer assignment is critical to peer review systems, such as peer-reviewed research conferences or peer-reviewed funding applications, and its effectiveness is a deep concern of all academics. However, there are some problems in existing peer review systems during reviewer assignment. For example, some of the reviewers are much more stringent than others, leading to an unfair final decision, i.e., some submissions (i.e., papers or applications) with better quality are rejected. In this paper, we propose a context-aware reviewer assignment for trust enhanced peer review. More specifically, in our approach, we first consider the research area specific expertise of reviewers, and the institution relevance and co-authorship between reviewers and authors, so that reviewers with the right expertise are assigned to the corresponding submissions without potential conflict of interest. In addition, we propose a novel cross-assignment paradigm, and reviewers are cross-assigned in order to avoid assigning a group of stringent reviewers or a group of lenient reviewers to the same submission. More importantly, on top of them, we propose an academic CONtext-aware expertise relevanCe oriEnted Reviewer cross-assignmenT approach (CONCERT), which aims to effectively estimate the “true” ratings of submissions based on the ratings from all reviewers, even though no prior knowledge exists about the distribution of stringent reviewers and lenient reviewers. The experiments illustrate that compared with existing approaches, our proposed CONCERT approach can less likely assign more than one stringent reviewers or lenient reviewers to a submission simultaneously and significantly reduce the influence of ratings from stringent reviewers and lenient reviewers, leading to trust enhanced peer review and selection, no matter what kind of distributions of stringent reviewers and lenient reviewers are.

## Introduction

The main aim of research conferences is to share research progress and novel findings among scholars and promote the growth of the whole academic disciplines. To this end, peer review systems have been introduced in conferences to guarantee the adequate quality of publications [1]. For the same reason, peer review systems have been introduced in funding applications.

A typical peer review process consists the following stages:

**Stage 1 (submission)**: Authors submit their proposed submissions (i.e., paper(s) or application(s)) to the peer review system of a relevant conference or a funding organization.

**Stage 2 (allocation and review)**: Before peer review process starts, each submission to be reviewed is allocated to a few reviewers (usually at least 3 reviewers) who are asked to make independent recommendations on whether the reviewed submission can be accepted or not [2].

**Stage 3 (decision making of organizers)**: In light of collected peer reviews, a decision is eventually made by organizers, on the acceptance of the reviewed submissions [2].

Peer review process is very important to the quality control of conferences and funding applications, and even further to the development of academic disciplines. Its effectiveness is a deep concern of all academics, as every scholar’s career is significantly influenced by it [3].

In general, there are some criticisms for existing peer review systems, which have been extensively discussed in [1, 2, 4]. These existing problems, such as reviews are unobjective and superficial, reviewers are stringent or lenient, and the review process involves too much work for reviewers, seriously blemish the reputation of conferences or funding organizations, lower their impact, slow down the innovation process, and frustrate both authors and reviewers [5].

In order to improve the quality of peer review process, a number of approaches have been proposed, focusing on different stages [1, 2, 5, 6]. For example, regarding Stage 1, Dittrich [5] proposes a peer review process to publish a paper section by section, and finally combine them together. Regarding Stage 2, Mimno and McCallum [6] propose that the expertise of each reviewer can be described by a distribution over topics, and then they rank reviewers based on the likelihood of the topics in a given paper under a reviewer’s distribution. While these approaches provide valuable solutions, some problems remain open during peer review process.

**P1** If a reviewer has conflict of interest with one of the authors of a submission, such as they have worked in the same institution (e.g., former colleagues) or they have co-authorship, the credibility of the review is doubtful.
**P2** As different reviewers usually have different understanding of the same review criteria due to the differences in their expertise, it is very likely that they give different ratings to the same submission assigned to them. Although in some existing peer review systems (e.g., AAAI and AAMAS) online discussion and panel meeting can be organized and partially solve this problem, no proper mechanism exists for automatically dealing with the disagreement between different ratings from reviewers.
**P3** As pointed out in Cognitive Science, reviewers have consistent tendency in reviews especially during a short time period [1, 7]. Thus, some reviewers are stringent giving low ratings while some other reviewers are lenient giving high ratings. If the group of reviewers assigned to a paper are stringent or lenient, aggregating such ratings directly leads to bias in making a final decision on the acceptance or rejection of a submission.
  In fact, in most existing peer review systems, it has been assumed that there is neither stringent reviewers nor lenient reviewers, and the obtained reviews are aggregated directly to estimate the ‘true’ rating of a submission. However, this assumption can hardly hold in real world.
**P4** For many peer review systems, reviewer set change significantly from year to year (For example, only 16.1% of all 540 reviewers for AAAI 2012 conference were recruited as the reviewers for AAAI 2013 conference). Hence, it is difficult to establish “reviewer reputation” for all reviewers to determine whether a reviewer is stringent or lenient according to his/her review history (If some reviewers can be determined to be stringent or lenient beforehand, both a stringent reviewer and a lenient reviewer should be assigned to a submission to reduce the bias when estimating the ‘true’ rating of the submission. The rest reviewers will be assigned with our proposed approach. In this paper we consider the most complicated case in which there is no pre-existing knowledge about the distribution of stringent reviewers and lenient reviewers.). This makes it very difficult to aggregate the ratings from both stringent reviewers and lenient reviewers to estimate the ‘true’ rating of a submission under review.
**P5** During reviewer assignment, there are usually constraints on both the number of reviewers assigned to each submission and the number of submissions allocated to each reviewer. In addition, it is necessary to minimize the bias caused by stringent reviewers and lenient reviewers. Therefore, context-aware reviewer assignment for trust enhanced review becomes a 0-1 integer programming problem with constraints, which is NP-complete [8].

When taking all these questions into account, it is very difficult for organizers, such as the conference program committee (PC) chairs, to manually assign reviewers to proper submissions, especially when the size of data set of submissions and reviewers is large. Though a double-blind peer review process (such as the one adopted in AAAI) can solve the first above-mentioned problem, the rest ones remain open.

In this paper, we propose an academic CONtext-aware expertise relevanCe oriEnted Reviewer cross-assignmenT approach (CONCERT), with the following characteristics and contributions.

1. The academic contexts of reviewers have been taken into account in our proposed reviewer assignment approach, which includes the research area specific expertise of reviewers, the institution relevance and the co-authorship relevance between reviewers and authors (to solve the above problem P1), and the expertise relevance between reviewers and submissions to be reviewed.
2. A novel cross-assignment paradigm is proposed for reviewer assignment (to solve the above problem P2). It aims to avoid assigning multiple stringent reviewers or lenient reviewers to a submission, and minimize the bias in reviews, even though no prior knowledge exists about the distribution of stringent reviewers and lenient reviewers (to solve the above problem P3 & P4).
3. Our proposed CONCERT approach is based on cross-assignment, and it aims to maximally eliminate the influence of ratings from stringent reviewers and lenient reviewers, and obtains objective review results (to solve the above problem P5). With the experimental results, we can observe that the CONCERT approach can lead to a much less variance of estimation bias than existing peer assignment approaches, though lenient reviewers or stringent reviewers have not be identified explicitly beforehand.

This paper is organized as follows. We firstly review existing studies on peer review. Then, a novel reviewer assignment approach CONCERT is proposed to reduce the influence of lenient reviewers and stringent reviewers. After that experimental results are presented and analyzed. Finally our work is concluded in this paper.

## Related Work

Although exiting peer review systems generally promote the growth of the whole academic disciplines, there are a lot of criticisms for them, which have been intensively discussed in a variety of scientific communities [1–4]. Now we summarize some typical problems from different perspectives. 1) From the perspective of authors, reviews could be unobjective, superficial, of low quality, or based on half-read papers [5]. 2) From the perspective of a reviewer, he/she is usually overloaded in the review process, with a lot of papers to be reviewed in a short time period. Some approaches, such as review processes involving author responses, review panels and in-person program committee meetings, are helpful to make objective decisions on the acceptance of the submissions; but they also require more work from reviewers [3]. It will be much better to have automatic process to significantly reduce time consumption. 3) From the perspective of program chairs, though it is possible to know who are stringent reviewers or lenient reviewers, it is very difficult to rule out all the stringent reviewers and lenient reviewers before reviewer assignment [4].

In order to improve the quality of peer review, a number of approaches have been proposed, focusing on different stages in the peer review process.

Regarding Stage 1 in the peer review process introduced in the Introduction Section, the improvement mainly focuses on shortening publication time and accelerating innovation process [5, 9]. In [5], Dittrich proposes a paper review process to publish a paper section by section, and finally combine them together. This system comes with a problem that it is hard to evaluate the contribution of research from just a small part of a paper. In [9], Perakakis et al. abandon the existing paper review process, suggest to publish the paper directly on the Web, and ask other scholars to post their online comments under the paper. However, in this model, there is no prior paper review for quality control and it may cause flooding of publications.

Regarding Stage 2 in the peer review process, in order to enhance the objectiveness and accuracy of reviews, reviewers are assigned to the submissions according to their related expertise. In [10], Benferhat and Lang take the assumption that both reviewers and authors come from the same research community, and all the topics of papers and all the expertise of reviewers can be described by key words selected from the same set. Then they focus on solving the optimization problem of matching papers and reviewers. In [6], Mimno and McCallum focus on a general range of the topics of papers and the expertise of reviewers. In their model, the expertise of each reviewer can be described by a distribution over topics, and then reviewers are ranked based on the likelihood of the topics in a given paper under the reviewer’s expertise distribution.

Regarding Stage 3 in the peer review process, in order to enhance the objectiveness of acceptance, ratings from reviewers are analyzed to settle the disagreement between different reviewers. Wood et al. [1] analyze the paper review system adopted in the 2001 and 2002 UK Academy for Information Systems conferences, introduce a concept “reliability” to estimate the probability of making errors when combining different reviews, and discuss some of the implications of reliability for authors, program chairs and development of academic knowledge. A high reliability means the ratings given by reviewers to a paper are similar to each other, while a low reliability means the ratings are quite different from each other. However, in [11], more general studies on reliability are introduced based on a total of 19443 paper reviews, which show that a high level reliability is considered less trustworthy than those with a low level reliability. Hence, the effectiveness of reliability in existing peer review systems is quite limited.

Although the above literature has addressed various aspects in peer review, there are still some important open problems as introduced in Introduction Section. This paper focuses on the solution to these open problems in peer review, which can be adopted by the organizers of a conference to improve the overall quality of accepted papers, especially when there are a large number of submissions in the conference.

## Academic Context-Aware Reviewer Assignment Approach

In this section, we will first introduce some important academic contexts [12], including the research area specific expertise of a reviewer, the institution relevance and the co-authorship relevance between reviewers and authors, and the expertise relevance between reviewers and submissions, all of which should be taken into account in reviewer assignment. Taking these academic contexts into account, we propose a reviewer assignment approach CONCERT, towards solving all the five problems in existing peer review systems as pointed out in Introduction Section.

### Academic Context Analysis

In this section, we present three types of academic contexts, including the academic expertise of reviewers, conflicts of interest between reviewers and authors, and the expertise relevance between reviewers and submissions, which have the most important influence on providing an accurate rating to a submission under review.

#### Academic Expertise of Reviewers

In real applications, we usually prefer to trust the suggestions from a reviewer with higher academic expertise in peer review systems. Although this controversial rule has been widely discussed, it is still widely adopted as there is no much better choice.

In the literature, the research area specific *h*-index has been proposed and calculated to estimate the academic expertise of a reviewer, which measures both the productivity and impact of a published paper of a scholar in a specific research area with the following Definition 1 [13]. Although variations of *h*-index have been proposed to take into account some of its perceived shortcomings [14, 15], there is no general consensus yet so far that any other single number bibliometric indicator is clearly preferable to *h*-index [13]. More specifically, *h*-index is evaluated based on the number of the scholar’s most cited papers and the number of citations that they have received in other publications in a specific research area, which is intended to measure simultaneously the quality and quantity of published research papers in the area.

**Definition 1** A scholar has a research area specific index *h* if *h* of his/her *N*_*p*_ papers have at least *h* citations each in a specific research area, and the rest *N*_*p*_ − *h* papers have no more than *h* citations each [13].

With the above definition, the research area specific *h*-index can be determined based on a citation database as in [16], such as Google Scholar, Scopus or Web of Knowledge. For example, the *h*-index of a scholar can be found online at Google Citations or ArnetMiner (http://arnetminer.org/). As different research fields use different numbers of citations as thresholds to determine the most cited papers, an *h*-index can be normalized by a simple rescaling factor to a specific area to obtain the research area specific *h*-index, such as the one in artificial intelligence or machine learning [15]. In the proposed system, the research area specific *h*-index can be obtained in PC invitations.

#### Conflicts of Interest Between Reviewers and Authors

According to the studies about negative outgroup bias in Cognitive Science [17], if a reviewer believes that an author belongs to his/her group, he/she will tend to give a better review. In order to describe this type of conflicts of interest (CoI) between a reviewer and an author of a submission to be reviewed, we introduce the *institution relevance* and the *co-authorship relevance* between a reviewer and an author of a submission to be reviewed respectively, which are the most prevalent CoI existing in peer review.

*Institution relevance* between a reviewer and one of the authors is to describe the fact that the institution the reviewer has worked in is the same as the institution of one of the authors of a submission to be reviewed (e.g., colleagues) (The academic contexts of a reviewer (e.g., working experience) can be obtained in PC invitations.).

*Co-authorship relevance* between a reviewer and one of authors means that they have co-authored at least one publication, research funding application or other academic activities.

In a double blind conference peer review process adopted in a number of prestigious conferences, there is no need to consider these types of CoIs, including both institution relevance and co-authorship relevance, as they have already been filtered out naturally. However, in other peer review systems which are the majority, they should be avoided explicitly during reviewer assignment.

### The Influence of Lenient Reviewers and Stringent Reviewers in Existing Peer Review Systems

**Fig 1:**
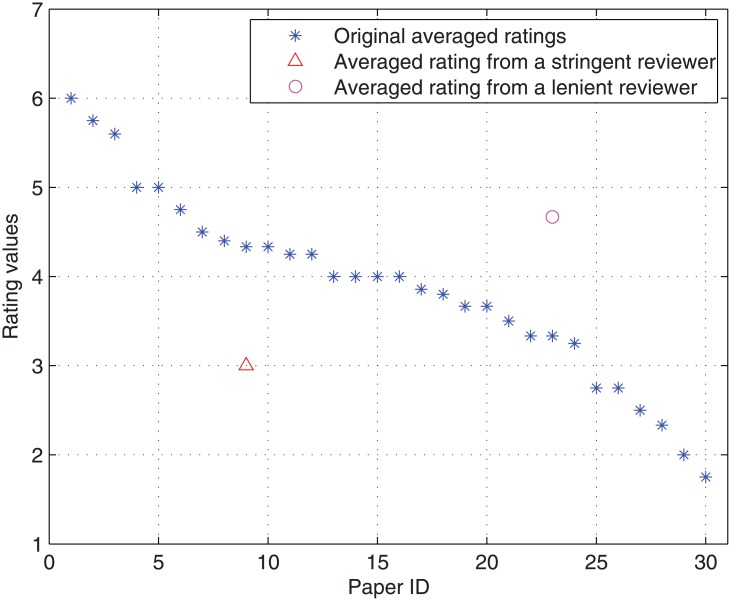
The influence of lenient reviewers and stringent reviewers in the peer review system of ATC-2011.

In existing peer review systems, the average or the weighted average of ratings of a submission is usually taken as the most important evidence to decide whether to accept this submission or not, where the weight can be determined by the expertise or expertise relevance of the reviewer. However, this is not rigorous enough. Let us take the peer review system of the 8th International Conference on Autonomic and Trusted Computing (ATC-2011 (http://cse.stfx.ca/∼atc2011/)) as an example and illustrate the reasons in two cases (The related data set from ATC-2011 can be downloaded from the Web, which can be accessed from http://socialysis.org/data/dataset/dataset/a2a9ba70-cb89-4a56-bf1b-5d8723c76734). In the ATC-2011 peer review system, each rating is an integer in {1, 2, 3, 4, 5, 6, 7}, with the corresponding linguistic interpretation as {Strong Reject, Reject, Weak Reject, Neutral, Weak Accept, Accept, Strong Accept}. The obtained average ratings from reviewers in this conference are listed in Fig 1.

**Case 1**: Let’s take paper *P*_9_ in Fig 1 as an example. If one of the reviewers assigned to *P*_9_ is stringent, he/she may give a rating 2 instead of 6 for this paper. Then the average rating of *P*_9_ changes from 4.33 to 3. This results in the downgrade of *P*_9_ from top 9 to top 24 out of 30 papers (i.e., downgrade from top 30% to top 80%) (see Fig 1), and the paper becomes rejected rather than accepted.

**Case 2**: Let’s take paper *P*_23_ in Fig 1 as an example. If one of the reviewers assigned to *P*_23_ is lenient, he/she may give a rating 6 instead of 2 for this paper. Then the average rating of *P*_23_ changes from 3.33 to 4.67. This results in the upgrade of *P*_23_ from top 23 to top 7 out of 30 papers (i.e., upgrade from top 76.67% to top 23.33%) (see Fig 1), and paper *P*_23_ becomes accepted rather than rejected.

From the above two cases, we can conclude that it leads to bias when using the rating averages directly to accept or reject a reviewed submission, due to the existence of lenient reviewers and stringent reviewers, especially when no prior knowledge exists about the distribution of stringent reviewers and lenient reviewers. Towards solving this outstanding problem, it is necessary to propose a novel reviewer assignment approach in order to avoid assigning stringent reviewers or lenient reviewers together to one submission.

Firstly, we consider the expertise of reviewers and the CoIs between reviewer and authors, and propose an academic context-aware reviewer cross-assignment (CAREER) approach. Then, in addition to the CAREER approach, we further take into account the expertise relevance between reviewers and submissions to be reviewed, and propose an academic context-aware expertise relevance oriented reviewer cross-assignment (CONCERT) approach.

### The CAREER Approach

With the academic contexts presented in the last few sections, towards reducing the influence of lenient reviewers and stringent reviewers, in this section, we propose an academic context-aware cross-assignment based reviewer assignment (CAREER) approach.

#### Principles and Strategies in the CAREER Approach

In this section, we propose the following principles and strategies which will be adopted in the CAREER approach:

**Principle 1**: We usually prefer trusting a reviewer with a higher research area specific *h*-index value.

**Principle 2**: If there is a type of CoI between the reviewer *R*_*j*_ and one of the authors of the submission *P*_*i*_, then *R*_*j*_ cannot be assigned to *P*_*i*_.

**Principle 3**: A reviewer *R*_*j*_ can be assigned to a submission up to once.

**Principle 4**: The number of reviewers assigned to a submission is no more than the maximal number *m*_*max*_ of reviewers assigned to a submission; the number of submissions allocated to a reviewer is no more than the maximal number *n*_*max*_ of submissions allocated to a reviewer.

**Principle 5**: If a reviewer *R*_*j*_ has been assigned to both submission *P*_*h*_ and submission *P*_*l*_, then there is an edge between *P*_*h*_ and *P*_*l*_ with the weight of 1, i.e., the distance *d*(*P*_*h*_, *P*_*l*_) = 1 in the graph (named as assignment graph). If reviewers *R*_*j*1_ and *R*_*j*2_ has been assigned to both submission *P*_*h*_ and submission *P*_*l*_, then we have *d*(*P*_*h*_, *P*_*l*_) = 0 in the assignment graph.

Based on Principle 5, we have the following definition first:

**Definition 2** With Dijkstra’s shortest path algorithm [18, 19], the distance between the submission *P*_*i*_ and the submission *P*_*j*_ can be determined by the shortest path between them. When the summation of distances of all submissions in the assignment graph is maximized, we call that submissions have been cross-allocated, or reviewers have been cross-assigned.

**Fig 2:**
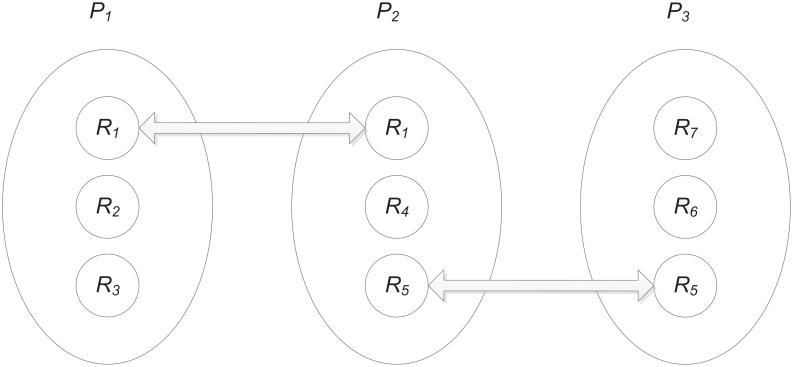
An example for the definition about distance between submissions.

Now let’s introduce a small example to illustrate the definition about distance between submissions, which is depicted in Fig 2. According to Principle 5, as *R*_1_ has been assigned to both *P*_1_ and *P*_2_, then we have *d*(*P*_1_, *P*_2_) = 1. Meanwhile, as *R*_5_ has been assigned to both *P*_2_ and *P*_3_, then we have *d*(*P*_2_, *P*_3_) = 1. Then, following Definition 2, we have *d*(*P*_1_, *P*_3_) = 2.

According to Definition 2, no matter which reviewer is stringent or lenient, submissions with reviewers have the maximized summation of distance to each other. When assigning a reviewer to a submission, a larger summation of distance means that it is the less likely that more than one stringent reviewers or lenient reviewers are assigned to a submission simultaneously; a smaller summation of distance means that it is the more likely that more than one stringent reviewers or lenient reviewers are assigned to a submission simultaneously. The more details about distance and cross-assignment will be introduced later in the next subsection.

**Strategy 1**: In order to minimize the influences caused by stringent reviewers and lenient reviewers, reviewers should be cross-assigned to the submissions. This reduces the possibility that more than one stringent reviewers or lenient reviewers are assigned to a submission simultaneously, and it subsequently minimizes the *estimation bias*, which is the expected value of the squared deviation from the ‘true’ rating because of stringent/lenient reviewers. Hence, the cross-assignment based approach can lead to a minimal estimation bias no matter which reviewer is stringent or lenient.

**Strategy 2**: In this paper, we cross-assign reviewers with a proposed greedy algorithm, i.e., an unassigned reviewer is assigned to a submission that has the largest summation of distance to other submissions without any CoI existing between this reviewer and the submissions’ authors.

In addition to the above Strategy 1 and Strategy 2, we have the following strategies particularly for the CAREER approach.

**Strategy 3**: Based on Principle 1, if a reviewer has a higher research area specific *h*-index value, during reviewer assignment, he/she will be assigned later. This will minimize the estimation bias caused by stringent reviewers or lenient reviewers.

**Strategy 4**: Based on Principle 1 and the fact that the research area specific *h*-index can be used to estimate the expertise of a reviewer, in the CAREER approach, we use the research area specific *h*-index as a weight to estimate the ‘true’ rating of the submission by the weighted average of ratings.

#### Cross-Assignment

Now let us illustrate the general idea of cross-assignment in our approaches with an example.

Firstly, we explain what is a lenient reviewer and what is a stringent reviewer. Suppose that there is a ‘true’ rating which accurately represents the academic quality of each submission [1, 20]. Then suppose the ‘true’ ratings for submissions *P*_1_ and *P*_2_ are ${\tilde{r}}_{1}=3$ and ${\tilde{r}}_{2}=6$ respectively.

**Fig 3:**
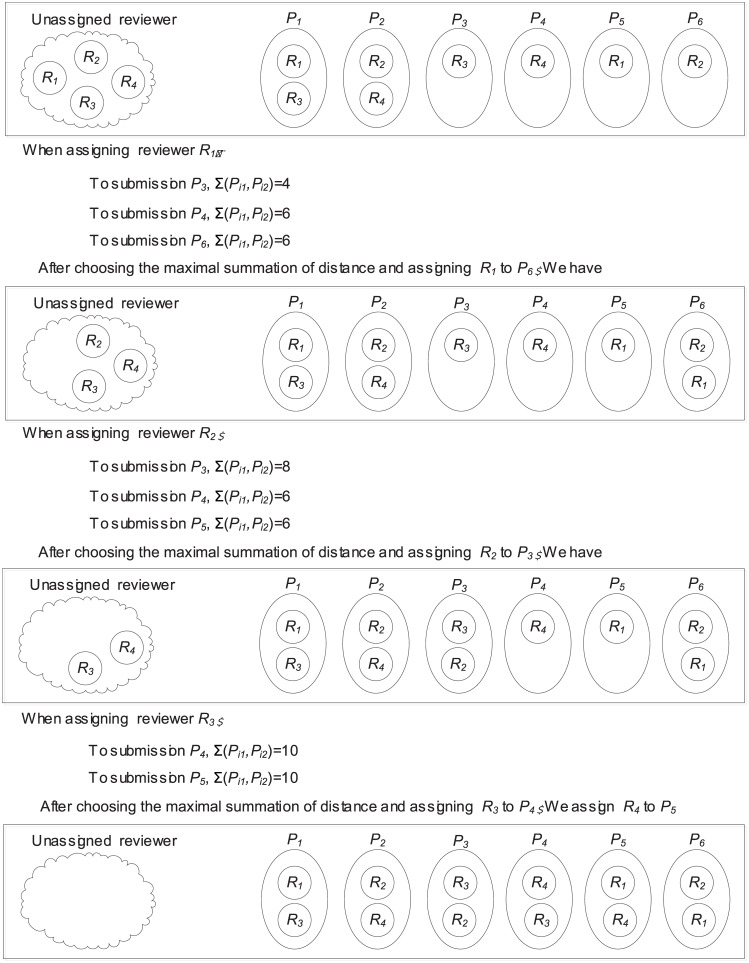
An example for the process of cross-assignment.

1. For a stringent reviewer, he/she would like to give lower ratings, e.g., *r*_1_ = 2 and *r*_2_ = 3;
2. For a lenient reviewer, he/she would like to give higher ratings, e.g., *r*_1_ = 4 and *r*_2_ = 7;

In this example, we consider the reviewer assignment case listed in Table 1 where reviewers *R*_1_, *R*_2_, *R*_3_ and *R*_4_ are to be assigned. There are totally 9 different reviewer assignments (a)–(i) depicted in Table 1. When assigning a reviewer *R*_*i*_ (*i* = 1, …, 4), *R*_*i*_ is assigned to the submission with the maximal summation of distance and the summation of distance is listed in brackets for each method in Table 1 (a)–(i). For example, as for reviewer assignment (a), *R*_2_ is assigned to *P*_5_ with the distance 8. According the above definition of cross-assignment, reviewer assignment (a) and reviewer assignment (g) have the largest summation of distance and they are the cases where reviewers are cross-assigned. The corresponding detailed process of cross-assignment is illustrated in Fig 3.

**Table 1:** The example about cross-assignment.

	Submissions	*P*_1_	*P*_2_	*P*_3_	*P*_4_	*P*_5_	*P*_6_
reviewer assignment case	Reviewer	*R*_1_	*R*_2_	*R*_3_	*R*_4_	*R*_1_	*R*_2_
	Reviewer	*R*_3_	*R*_4_				
reviewer assignment (a)	Reviewer	*R*_1_	*R*_2_	*R*_3_	*R*_4_	*R*_1_	*R*_2_
	Reviewer	*R*_3_	*R*_4_	*R*_4_(12)	*R*_1_(6)	*R*_2_(8)	*R*_3_(10)
reviewer assignment (b)	Reviewer	*R*_1_	*R*_2_	*R*_3_	*R*_4_	*R*_1_	*R*_2_
	Reviewer	*R*_3_	*R*_4_	*R*_1_(4)	*R*_2_(4)	*R*_3_(2)	*R*_4_(0)
reviewer assignment (c)	Reviewer	*R*_1_	*R*_2_	*R*_3_	*R*_4_	*R*_1_	*R*_2_
	Reviewer	*R*_3_	*R*_4_	*R*_2_(8)	*R*_1_(6)	*R*_3_(8)	*R*_4_(8)
reviewer assignment (d)	Reviewer	*R*_1_	*R*_2_	*R*_3_	*R*_4_	*R*_1_	*R*_2_
	Reviewer	*R*_3_	*R*_4_	*R*_2_(8)	*R*_1_(6)	*R*_4_(8)	*R*_3_(8)
reviewer assignment (e)	Reviewer	*R*_1_	*R*_2_	*R*_3_	*R*_4_	*R*_1_	*R*_2_
	Reviewer	*R*_3_	*R*_4_	*R*_1_(4)	*R*_2_(4)	*R*_4_(8)	*R*_3_(6)
reviewer assignment (f)	Reviewer	*R*_1_	*R*_2_	*R*_3_	*R*_4_	*R*_1_	*R*_2_
	Reviewer	*R*_3_	*R*_4_	*R*_1_(4)	*R*_3_(8)	*R*_2_(6)	*R*_4_(8)
reviewer assignment (g)	Reviewer	*R*_1_	*R*_2_	*R*_3_	*R*_4_	*R*_1_	*R*_2_
	Reviewer	*R*_3_	*R*_4_	*R*_2_(8)	*R*_3_(10)	*R*_4_(12)	*R*_1_(6)
reviewer assignment (h)	Reviewer	*R*_1_	*R*_2_	*R*_3_	*R*_4_	*R*_1_	*R*_2_
	Reviewer	*R*_3_	*R*_4_	*R*_4_(8)	*R*_2_(6)	*R*_3_(6)	*R*_1_(6)
reviewer assignment (i)	Reviewer	*R*_1_	*R*_2_	*R*_3_	*R*_4_	*R*_1_	*R*_2_
	Reviewer	*R*_3_	*R*_4_	*R*_4_(8)	*R*_3_(8)	*R*_2_(6)	*R*_1_(6)

**Fig 4:**
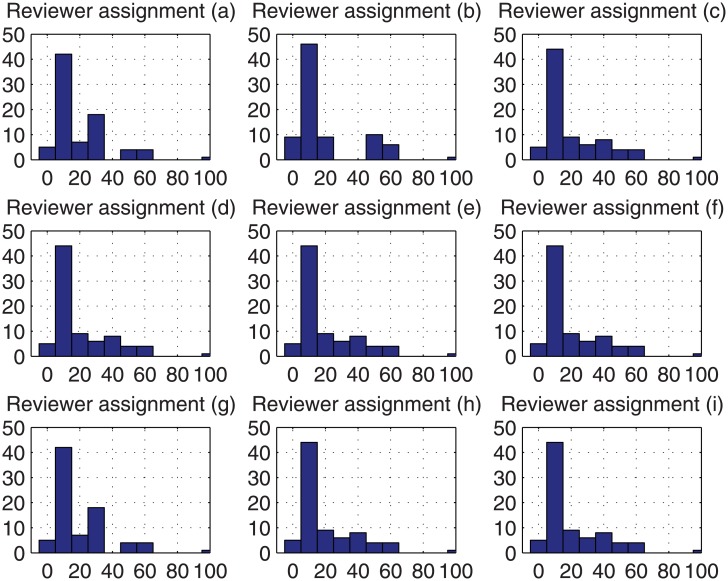
Histogram of estimation bias.

Without loss of generality, for each reviewer we consider three different situations, including (1) the reviewer is lenient and provides ratings with 2 more than the ‘true’ rating, (2) the reviewer provides the ‘true’ rating, and (3) the reviewer is stringent and provides ratings with 1 less than the ‘true’ rating. Then for each reviewer assignment in Table 1 (a)–(i), we can compute the estimation bias, which is depicted by the histogram in Fig 4 with different kinds of distributions of stringent reviewers and lenient reviewers. The corresponding variances of estimation bias for each reviewer assignment in Table 1 (a)–(i) are 275.7, 393.3, 314.9, 314.9, 314.9, 314.9, 275.7, 314 and 314.9 respectively.

We can observe that in each of case (a) and case (g), the variance is minimal, and this confirms that the cross-assignment leads to the smallest variance of estimation bias, no matter what kind of distributions of stringent reviewers and lenient reviewers are, i.e., the cross-assignment leads to a stable estimated rating bias, even though no prior knowledge exists about the distribution of stringent reviewers and lenient reviewers.

During reviewer assignment, there are constraints on both the number of reviewers assigned to each submission and the number of submissions allocated to each reviewer. Hence, as a knapsack problem, reviewer assignment with constraints is a 0-1 integer programming problem which is NP-complete [8]. Therefore, in existing reviewer assignment approaches [8], greedy algorithms have been used to gradually assign a reviewer to a submission. In our proposed greedy approach CAREER, in particular, during reviewer assignment, *R*_*j*_ is assigned to the submission which has the maximal summation of distance *d* to other submissions without any CoI between *R*_*j*_ and authors of the submission, aiming at avoiding assigning a group of stringent reviewers or a group of lenient reviewers to a submission.

#### The Proposed CAREER Approach

Our proposed CAREER approach (Algorithm 1) works as follows.

**Algorithm 1:** The CAREER Approach

**Data**: Submission set {*P*_*i*_∣*i* ∈ [1, *n*]}; Reviewer set {*R*_*j*_∣*j* ∈ [1, *m*]}; The maximal number *n*_*max*_ of submissions allocated to a reviewer in the system; The maximal number *m*_*max*_ of reviewers assigned to a submission in the system.

**Result**: Assigned reviewer set $\left\{R_{k}^{\left(P_{i}\right)}\right\}$ for each submission.

1 **begin**

2 **if** *m* * *n*_max_ > *n* * *m*_max_ **then**

3  *n*_*max*_ = ⌊*n* * *m*_*max*_/*m*⌋;

4 **else**

5  *m*_*max*_ = ⌊*m* * *n*_*max*_/*n*⌋;

6 **end**

7 rearrange {*R*_*j*_∣*j* ∈ [1, *m*]} according to research area specific *h*-index values;

8 **for** *R*_*h*_*i*__ ∈ {*R*_*j*_∣*j* ∈ [1, *m*]} **do**

9  **for** *R*_*h*_*j*__ ∈ {*R*_*j*_∣*j* ∈ [1, *m*]} **do**

10   *d*(*R*_*h*_*i*__, *R*_*h*_*j*__) ⇐ ∞;

11  **end**

12 **end**

13 initialize the reviewer set $\{R_{k}^{\left(P_{i}\right)}\}⇐\emptyset$ and $n_{R}^{\left(P_{i}\right)}⇐\text{size}\{R_{k}^{\left(P_{i}\right)}\}$;

14 initialize the submission set $\{P_{h}^{\left(R_{j}\right)}\}⇐\emptyset$ for each reviewer;

15 **for** *l* ∈ [1, *n*_max_] **do**

16  **for** *R*_*j*_ ∈ {*R*_*j*_∣*j* ∈ [1, *m*]} **do**

17   calculate $d(\{P_{h}^{\left(R_{j}\right)}\},\{P_{i}\}-\{P_{h}^{\left(R_{j}\right)}\})$;

18   find the maximal *d* without CoI between *R*_*j*_ and authors of *P*_*i*_;

19   under *d*, assign *R*_*j*_ to that submission P*;

20   $\{P_{h}^{\left(R_{j}\right)}\}⇐\{P_{h}^{\left(R_{j}\right)}\}\cup P^{*}$;

21   $\{R_{k}^{\left(P^{*}\right)}\}⇐\{R_{k}^{\left(P^{*}\right)}\}\cup R_{j}$;

22   **for** *P*_*h*_*j*__ ∈ {*P*_*i*_∣*i* ∈ [1, *n*]} **do**

23    **for** *P*_*h*_*k*__ ∈ {*P*_*i*_∣*i* ∈ [1, *n*]} **do**

24     **if** $\{R_{k}^{\left(P_{h_{j}}\right)}\}\cap \{R_{k}^{\left(P_{h_{k}}\right)}\}\neq \emptyset$ **then**

25      $d(R_{k}^{\left(1\right)},R_{k}^{\left(2\right)})⇐1$;

26     **end**

27    **end**

28   **end**

29  **end**

30 **end**

31 **return** $\left\{R_{k}^{\left(P_{i}\right)}\right\}$;

32 **end**

**Step 1**: During reviewer assignment, consider the total number of submissions *n*, the maximal number of reviewers assigned to a submission *m*_*max*_, the total number of reviewers *m* and the maximal number of submissions allocated to a reviewer *n*_*max*_, with any three of these factors, the fourth factor in applications can be calculated. More specifically, if

$$\begin{array}{c} m*n_{max}>n*m_{max}, \end{array}$$ (1)

we have

$$\begin{array}{c} n_{max}=\left\lfloor \frac{n*m_{max}}{m}\right\rfloor ; \end{array}$$ (2)

otherwise,

$$\begin{array}{c} m_{max}=\left\lfloor \frac{m*n_{max}}{n}\right\rfloor \end{array}$$ (3)

(line 2-6 in Algorithm 1).

**Step 2**: According to Strategy 3, rearrange reviewers according to their research area specific *h*-index values, and assign reviewers with lower research area specific *h*-index firstly (line 7).

**Step 3**: Initialize the adjacent matrix with *n* vertices where the weight of the edge between reviewed submission *P*_*h*_*i*__ and reviewed submission *P*_*h*_*j*__ is

$$d\left(P_{h_{i}},P_{h_{j}}\right)=\infty \;\left(h_{i},h_{j}=1,\cdots ,n\right)$$

(*O*(*n* ^2^)) (lines 8–12).

**Step 4**: Initialize the set of reviewers $\left\{R_{k}^{\left(P_{i}\right)}\right\}$ assigned to submission *P*_*i*_ as empty, and initialize the set of submissions $\left\{P_{h}^{\left(R_{j}\right)}\right\}$ allocated to the reviewer *R*_*j*_ as empty (*O*(*n* + *m*)) (lines 13–14).

1. When allocating the reviewer *R*_*j*_,
  $$d(\left\{P_{h}^{\left(R_{j}\right)}\right\},\left\{P_{i}\right\}-\left\{P_{h}^{\left(R_{j}\right)}\right\})$$
  is calculated. Then according to Strategy 2, *R*_*j*_ is assigned to the submission $P^{*}\in \{P_{i}\}-\{P_{h}^{\left(R_{j}\right)}\}$ with the maximal *d* and without any CoI between *R*_*j*_ and the authors of P* (*O*((*n* + *m*_*max*_)*n* log *n*) (lines 16–21);
2. If the reviewer *R*_*j*_ has been assigned to both submission *P*_*h*_ and submission *P*_*l*_, then
  $$d(P_{h},P_{l})=1$$
  (*O*(*n*)) (lines 22–28);
3. All other reviewers can be assigned in the order from low research area specific *h*-index values to high research area specific *h*-index values, following the same procedure introduced in a) & b) (*O*(*m*)) (lines 16–29).

**Step 5:** The above process in Step 4 (denoted as a round) repeats until the number of allocated submissions for each reviewer reaches *n*_*max*_ (lines 15–30).

The CAREER approach (Algorithm 1) incurs a complexity of *O*((*n* + *m*_*max*_)*mnn*_*max*_ log *n*), where *n* is the total number of submissions, *m* is the total number of reviewers, *m*_*max*_ is the maximal number of reviewers assigned to a submission in the system, and *n*_*max*_ is the maximal number of submissions allocated to a reviewer in the system. As *m*_*max*_ and *n*_*max*_ are usually less than 10, the CAREER approach incurs a complexity of *O*(*mn* ^2^ log *n*).

### The CONCERT Approach

We usually prefer trusting a reviewer who has a higher expertise relevance on the topics of submissions [1], because he/she is able to understand the quality of submissions better, and then can write more objective reviews. As this important contextual factor has not been taken into account in the above CAREER approach, we propose a novel academic context-aware and expertise relevance oriented cross-assignment based reviewer assignment (CONCERT) approach in this section.

Firstly, let us illustrate how to technically evaluate expertise relevance between reviewers and submissions. In Semantic Web, the typical approaches for the topics matching between reviewers and submissions adopt Kullback-Leibler (KL) divergence value [8] to quantitatively evaluate the relevance between the expertise of a reviewer and the topics of a submission. More specifically, the KL-divergence value *D* between the expertise distribution *d*_*R*_*j*__ of a reviewer *R*_*j*_ and the topics distribution *d*_*P*_*i*__ of a submission *P*_*i*_ can be defined as

$$\begin{array}{c} D(d_{R_{j}}\left|\right|d_{P_{i}})=\sum_{x}d_{R_{j}}\left(x\right)\log\frac{d_{R_{j}}\left(x\right)}{d_{P_{i}}\left(x\right)}. \end{array}$$ (4)

Taking other contextual factors into account, we propose one more important principle and one more important strategy, which are considered during reviewer assignment in the proposed CONCERT approach.

**Principle 6**: The relevance between the expertise of a reviewer and the topics of a submission should be maximized, i.e., the KL-divergence value between the expertise distribution *d*_*R*_*j*__ of a reviewer *R*_*j*_ and the topics distribution *d*_*P*_*i*__ of a submission *P*_*i*_ should be maximized.

**Strategy 5**: Both the research area specific *h*-index *h*(*R*_*j*_) of a reviewer *R*_*j*_ and the expertise relevance *D*(*d*_*R*_*j*__∣∣*d*_*P*_*i*__) between reviewer *R*_*j*_ and submission *P*_*i*_ are used to estimate the expertise of the reviewer *R*_*j*_, which is helpful to provide a rating with less estimation bias from the ‘true’ rating of a submission. This is because the higher the *h*(*R*_*j*_), the more likely for us to trust reviewer *R*_*j*_ to give an accurate rating for the reviewed submission; the larger the *D*(*d*_*R*_*j*__∣∣*d*_*P*_*i*__), the better reviewer *R*_*j*_ will understand the quality of submission *P*_*i*_, and the more likely for for him/her to provide objective reviews. Hence, the larger *h*(*R*_*j*_)*D(*d*_*R*_*j*__∣∣*d*_*P*_*i*__), the more likely for reviewer *R*_*j*_ to give a rating which is the same as the ‘true’ rating for the reviewed submission.

The Principles 2-6 convert the reviewer assignment problem to a multi-objective programming problem, which is NP-complete [8]. Here we design a greedy algorithm CONCERT to gradually select reviewers and assign them to submissions. More specifically, during reviewer assignment, we adopt the following strategy.

**Strategy 6**: When assigning a reviewer *R*_*j*_ to a submission *P*_*i*_, we maximize the utility *U*(*R*_*j*_, *P*_*i*_) as follows

$$\begin{array}{ccc} U(R_{j},P_{i}) & = & \omega_{1}D(d_{R_{j}}\left|\right|d_{P_{i}})-\omega_{2}h\left(R_{j}\right)+\omega_{3}\sum d\left(\left\{P_{h}^{\left(R_{j}\right)}\right\},\left\{P_{i}\right\}-\left\{P_{h}^{\left(R_{j}\right)}\right\}\right) \end{array}$$ (5)

where *D*(*d*_*R*_*j*__∣∣*d*_*P*_*i*__) is the KL-divergence value [8] between the expertise distribution *d*_*R*_*j*__ of a reviewer *R*_*j*_ and the topics distribution *d*_*P*_*i*__ of a submission *P*_*i*_; *h*(*R*_*j*_) is the research area specific *h*-index value of the reviewer *R*_*j*_; $\sum d(\{P_{h}^{\left(R_{j}\right)}\},\{P_{i}\}-\{P_{h}^{\left(R_{j}\right)}\})$ is the summation of distance from other submissions allocated to the reviewer *R*_*j*_, which doesn’t have any CoI between *R*_*j*_ and the authors of submission *P*_*i*_. It is used to evaluate the degree of cross-assignment; *ω*_1_, *ω*_2_ and *ω*_3_ are the weights to control the relative importance of *D*(*d*_*R*_*j*__∣∣*d*_*P*_*i*__), *h*(*R*_*j*_) and $d(\{P_{h}^{\left(R_{j}\right)}\},\{P_{i}\}-\{P_{h}^{\left(R_{j}\right)}\})$, respectively.

Taking the Principles 2-6 and Strategies 5-6 into account, our proposed CONCERT approach (Algorithm 2) works as follows.

**Algorithm 2** The CONCERT Approach

**Data**: Submission set {*P*_*i*_∣*i* ∈ [1, *n*]}; Reviewer set {*R*_*j*_∣*j* ∈ [1, *m*]}; The maximal number *n*_*max*_ of submissions allocated to a reviewer in the system; The maximal number *m*_*max*_ of reviewers assigned to a submission in the system.

**Result**: Assigned reviewer set $\left\{R_{k}^{\left(P_{i}\right)}\right\}$ for each submission.

1 **begin**

2 **if** *m* * *n*_max_ > *n* * *m*_max_ **then**

3  *n*_*max*_ = ⌊*n* * *m*_*max*_/*m*⌋;

4 **else**

5  *m*_*max*_ = ⌊*m* * *n*_*max*_/*n*⌋;

6 **end**

7 **for** *R*_*h*_*i*__ ∈ {*R*_*j*_∣*j* ∈ [1, *m*]} **do**

8  **for** *R*_*h*_*j*__ ∈ {*R*_*j*_∣*j* ∈ [1, *m*]} **do**

9   *d*(*R*_*h*_*i*__, *R*_*h*_*j*__) ⇐ ∞;

10  **end**

11 **end**

12 initialize the reviewer set $\{R_{k}^{\left(P_{i}\right)}\}⇐\emptyset$ and $n_{R}^{\left(P_{i}\right)}⇐\text{size}\{R_{k}^{\left(P_{i}\right)}\}$;

13 initialize the submission set $\{P_{h}^{\left(R_{j}\right)}\}⇐\emptyset$ for each reviewer;

14 **for** *l* ∈ [1, *m* * *n*_max_] **do**

15  calculate utility $U(\{P_{h}^{\left(R_{j}\right)}\},\{P_{i}\}-\{P_{h}^{\left(R_{j}\right)}\})$;

16  find the maximal utility without CoI between *R*_*j*_ and the authors of *P*_*i*_;

17  under the utility, assign $R_{j}^{*}$ to submission P*;

18  $\{P_{h}^{\left(R_{j}^{*}\right)}\}⇐\{P_{h}^{\left(R_{j}^{*}\right)}\}\cup P^{*}$;

19  $\{R_{k}^{\left(P^{*}\right)}\}⇐\{R_{k}^{\left(P^{*}\right)}\}\cup R_{j}^{*}$;

20  **for** *P*_*h*_*j*__ ∈ {*P*_*i*_∣*i* ∈ [1, *n*]} **do**

21   **for** *P*_*h*_*k*__ ∈ {*P*_*i*_∣*i* ∈ [1, *n*]} **do**

22    **if** $\{R_{k}^{\left(P_{h_{j}}\right)}\}\cap \{R_{k}^{\left(P_{h_{k}}\right)}\}\neq \emptyset$ **then**

23     $d(R_{k}^{\left(1\right)},R_{k}^{\left(2\right)})⇐1$;

24    **end**

25   **end**

26  **end**

27 **end**

28 **return** $\left\{R_{k}^{\left(P_{i}\right)}\right\}$;

29 **end**

**Step 1**: Given the total number of submissions *n*, the maximal number of reviewers assigned to a submission *m*_*max*_, the total number of reviewers *m* and the maximal number of submissions allocated to a reviewer *n*_*max*_, with any three of above factors, the fourth factor can be calculated. More specifically, if

$$\begin{array}{c} m*n_{max}>n*m_{max}, \end{array}$$ (6)

we have

$$\begin{array}{c} n_{max}=\left\lfloor \frac{n*m_{max}}{m}\right\rfloor ; \end{array}$$ (7)

otherwise,

$$\begin{array}{c} m_{max}=\left\lfloor \frac{m*n_{max}}{n}\right\rfloor , \end{array}$$ (8)

(line 2-6 in Algorithm 2).

**Step 2**: Initialize the adjacent matrix with *n* vertices where the weight of the edge between the submission *P*_*h*_*i*__ and the submission *P*_*h*_*j*__ is

$$d\left(P_{h_{i}},P_{h_{j}}\right)=\infty \;\left(h_{i},h_{j}=1,\cdots ,n\right)$$

(*O*(*n* ^2^)) (lines 7–11).

**Step 3**: Initialize the set of assigned reviewers $\left\{R_{k}^{\left(P_{i}\right)}\right\}$ for submission *P*_*i*_ as empty, and initialize the set of allocated submissions $\left\{P_{h}^{\left(R_{j}\right)}\right\}$ for the reviewer *R*_*j*_ as empty (*O*(*n* + *m*)) (lines 12–13).

**Step 4**:

1. With Eq (5), the utility *U*(*R*_*j*_, *P*_*i*_) of reviewer *R*_*j*_ and submission *P*_*i*_ can be calculated. Find the maximal *U*(*R*_*j*_, *P*_*i*_) such that *P*_*i*_ has not been allocated to *R*_*j*_ and there is no CoI between *R*_*j*_ and *P*_*i*_. Then assign reviewer *R*_*j*_ to submission *P*_*i*_ (*O*((*n* + *m*_*max*_)*mn* log *n*) (lines 15–19);
2. If the reviewer *R*_*j*_ has been assigned to both submission *P*_*h*_ and submission *P*_*l*_, then
  $$d(P_{h},P_{l})=1$$
  (*O*(*n*)) (lines 20–26);
3. All the other reviewers can be assigned accordingly following the same procedure introduced in a) & b) until the number of allocated submissions for each reviewer reaches *n*_*max*_ or all the reviewers have been assigned (lines 14–27).

The CONCERT approach (Algorithm 2) incurs a complexity of *O*((*n* + *m*_*max*_)*m* ^2^ *nn*_*max*_ log *n*), where *n* is the total number of submissions, *m* is the total number of reviewers, *m*_*max*_ is the maximal number of reviewers assigned to a submission in the system, and *n*_*max*_ is the maximal number of submissions allocated to a reviewer in the system. As *m*_*max*_ and *n*_*max*_ are usually less than 10, the CONCERT approach incurs a complexity of *O*(*m* ^2^ *n* ^2^ log *n*).

## Experiments

We have conducted experiments to illustrate the effectiveness of our proposed CONCERT approach. In peer review systems, as it is hard to obtain the ‘true’ rating representing the academic quality of a submission, we have to use a synthetic data set rather than a real data set in our experiments.

In the experiments, the synthetic data set has *m* = 75 reviewers and *n* = 100 submissions. The maximal number of reviewers assigned to a submission in the peer review system is *m*_*max*_ = 3, and the maximal number of submissions allocated to a reviewer is *n*_*max*_ = 4. Hence, there are 300 ratings in total in this data set, as there are 75 reviewers and each reviewer provides 4 ratings.

As the ‘true’ rating of a submission is not directly relevant to evaluate the bias introduced by stringent/lenient reviewers, we focus on the difference between the ratings of a submission from stringent/lenient reviewers and the ‘true’ rating. Without loss of generality, we assume that the ‘true’ rating is in the set {1, 2, 3, 4, 5, 6, 7}, and and the difference between the ratings from stringent/lenient reviewers and the ‘true’ rating is in the set {−2, −1, 0, 1, 2}. In the experiments, we consider 1000 different cases about the distribution of difference between the ratings from stringent/lenient reviewers and the ‘true’ rating, and 12 of them are randomly selected and depicted in Fig 8. In this figure we can observe that the difference function converges to zero. This confirms the fact that if a reviewer has a higher research area specific *h*-index values, we usually prefer trusting them less likely to be a stringent reviewer or a lenient reviewer and the difference $\left(r_{k}^{\left(P_{i}\right)}-{r^{*}}^{\left(P_{i}\right)}\right)$ will be smaller.

### Experiment 1—Study on the ATC Review Data

In this experiment, we compare the CAREER approach with the reviewer assignment approach adopted in ATC-2011, to illustrate the CAREER approach can reduce the estimation bias caused by the influence from stringent reviewers and lenient reviewers.

As the expertise relevance between reviewers and submissions has not been taken into account in the CAREER approach, in this experiment, we use the weighted average ${\bar{r}}^{\left(P_{i}\right)}$ of ratings $\left\{r_{k}^{\left(P_{i}\right)}\right\}$ to estimate the ‘true’ rating of a submission *P*_*i*_,

$$\begin{array}{c} {\bar{r}}^{\left(P_{i}\right)}=\frac{\sum_{k=1}^{m_{max}}\omega_{k}^{\left(R_{j},P_{i}\right)}\cdot r_{k}^{\left(P_{i}\right)}}{\sum_{k=1}^{m_{max}}\omega_{k}^{\left(R_{j},P_{i}\right)}}, \end{array}$$ (9)

where the weight $\omega_{k}^{\left(R_{j},P_{i}\right)}=h(R_{j})$ is the research area specific *h*-index value of a reviewer *R*_*j*_, which is due to Strategy 4.

With all the ratings $\left\{r_{k}^{\left(P_{i}\right)}\right\}$ provided by reviewers and all the ‘true’ ratings {r*^(*P*_*i*_)^} for submissions {*P*_*i*_}, the influence caused by stringent reviewers and lenient reviewers can be quantitatively evaluated as the estimation bias as follows:

$$\begin{array}{ccc} B(\left\{r_{k}^{\left(P_{i}\right)}\right\},{r^{*}}^{\left(P_{i}\right)}) & = & \frac{\sum_{i=1}^{n}{\left({\bar{r}}^{\left(P_{i}\right)}-{r^{*}}^{\left(P_{i}\right)}\right)}^{2}}{n} \end{array}$$ (10)

$$\begin{array}{ccc} & = & \frac{\sum_{i=1}^{n}{\left(\frac{\sum_{k=1}^{m_{max}}\omega_{k}^{\left(R_{j},P_{i}\right)}\cdot r_{k}^{\left(P_{i}\right)}}{\sum_{k=1}^{m_{max}}\omega_{k}^{\left(R_{j},P_{i}\right)}}-{r^{*}}^{\left(P_{i}\right)}\right)}^{2}}{n}. \end{array}$$ (11)

In a good peer review system, the mean and the variance of estimation bias $B(\{r_{k}^{\left(P_{i}\right)}\},{r^{*}}^{\left(P_{i}\right)})$ are expected to be as less as possible.

In addition, in this experiment, we have considered the following case about experiment settings: as the research area specific *h*-index values of reviewers in ATC-2011 can be obtained in PC invitations, we only focus on the difference $\left(r_{k}^{\left(P_{i}\right)}-{r^{*}}^{\left(P_{i}\right)}\right)$ rather than the ‘true’ rating r*^(*P*_*i*_)^ of submission *P*_*i*_. This is due to the fact that

$$\begin{array}{ccc} B(\left\{r_{k}^{\left(P_{i}\right)}\right\},{r^{*}}^{\left(P_{i}\right)}) & = & \frac{\sum_{i=1}^{n}{\left(\frac{\sum_{k=1}^{m_{max}}\omega_{k}^{\left(R_{j},P_{i}\right)}\cdot r_{k}^{\left(P_{i}\right)}}{\sum_{k=1}^{m_{max}}\omega_{k}^{\left(R_{j},P_{i}\right)}}-{r^{*}}^{\left(P_{i}\right)}\right)}^{2}}{n} \end{array}$$ (12)

$$\begin{array}{ccc} & = & \frac{\sum_{i=1}^{n}{\left(\frac{\sum_{k=1}^{m_{max}}\omega_{k}^{\left(R_{j},P_{i}\right)}\cdot \left[\left(r_{k}^{\left(P_{i}\right)}-{r^{*}}^{\left(P_{i}\right)}\right)+{r^{*}}^{\left(P_{i}\right)}\right]}{\sum_{k=1}^{m_{max}}\omega_{k}^{\left(R_{j},P_{i}\right)}}-{r^{*}}^{\left(P_{i}\right)}\right)}^{2}}{n} \end{array}$$ (13)

$$\begin{array}{ccc} & = & \frac{\sum_{i=1}^{n}{\left(\frac{\sum_{k=1}^{m_{max}}\omega_{k}^{\left(R_{j},P_{i}\right)}\cdot \left(r_{k}^{\left(P_{i}\right)}-{r^{*}}^{\left(P_{i}\right)}\right)}{\sum_{k=1}^{m_{max}}\omega_{k}^{\left(R_{j},P_{i}\right)}}\right)}^{2}}{n}. \end{array}$$ (14)

**Fig 5:**
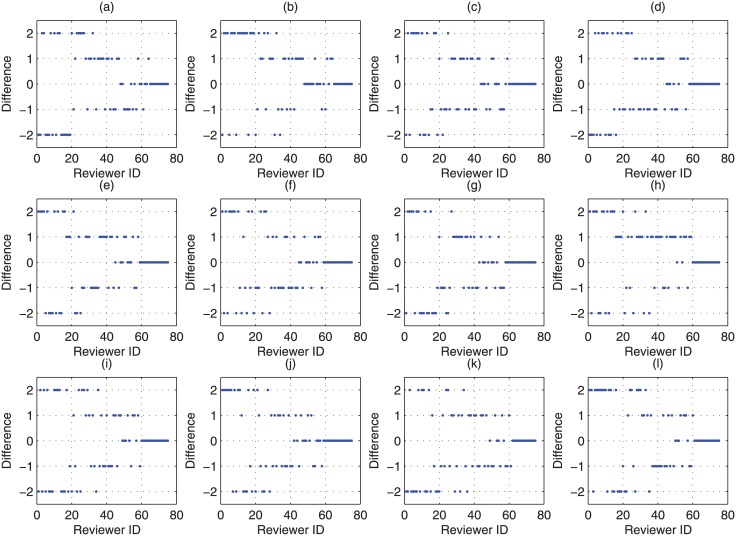
The estimation bias from the reviewer assignment approach adopted in ATC-2011 and the CAREER approach.

Here we randomly select 10^5^ different cases of the difference $\left(r_{k}^{\left(P_{i}\right)}-{r^{*}}^{\left(P_{i}\right)}\right)$, which belongs to {−2, −1, 0, 1, 2}. Both the reviewer assignment approach adopted in ATC-2011 and CAREER approach are applied to the above 10^5^ cases, 100 of them are randomly selected and plotted in Fig 5. With these 10^5^ cases, the mean and variance of estimation bias from the reviewer assignment approach adopted in ATC-2011 are 0.4077 and 0.0148 respectively. In contrast, the mean and variance of estimation bias from CAREER approach are 0.3431 and 0.0106 respectively. Hence, the mean and variance of results from CAREER approach are only 84.16% and 71.62% of that from the reviewer assignment approach adopted in ATC-2011. Therefore, the CAREER approach is better than the reviewer assignment approach adopted in ATC-2011 on the estimation bias reduction caused by stringent reviewers and lenient reviewers, though we assume that lenient reviewers or stringent reviewers cannot be identified explicitly beforehand.

### Experiment 2—Study on the CAREER Approach

In this experiment, we aim to illustrate that our proposed CAREER approach can reduce the influence caused by stringent reviewers and lenient reviewers, i.e., the variance of estimation bias can be reduced.

In this experiment, we compare the CAREER approach with a Monte Carlo based reviewer assignment approach. In the Monte Carlo based reviewer assignment approach, though the reviewers are assigned to the submissions randomly, Principles 2-4 are still followed.

The reason why we introduce the Monte Carlo based reviewer assignment approach is due to the following principle: the more times the Monte Carlo based reviewer assignment approach is executed, the more likely it can cover any result of all reviewer assignment approaches. Hence, after we have executed the Monte Carlo based reviewer assignment approach for sufficient times, we can analyze to what extent our proposed approach is superior to other reviewer assignment approaches.

In addition, in this experiment, we have considered the following cases about experiment settings.

**Fig 6:**
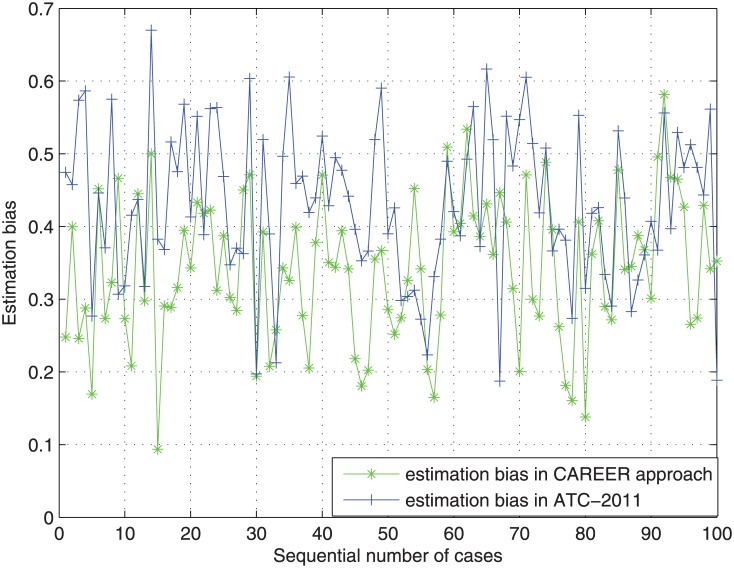
The distribution of the ‘true’ ratings for submission in experiments.

**Fig 7:**
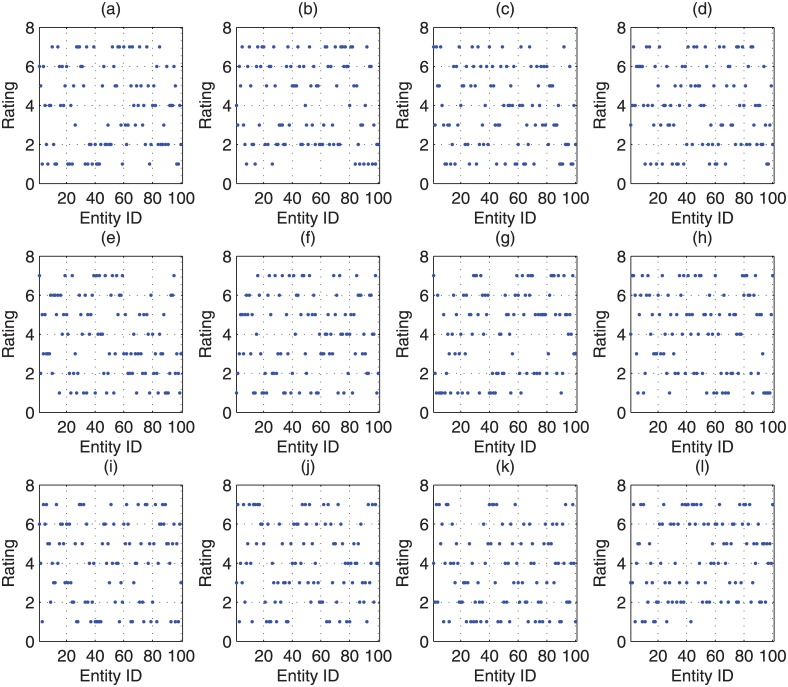
The distribution of the research area specific *h*-index for reviewers in experiments.

**Fig 8:**
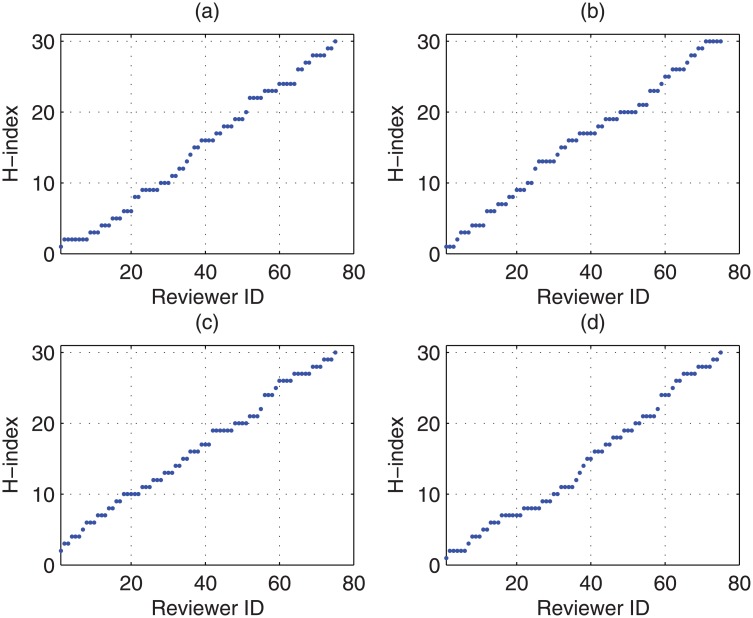
The distribution of difference between the provided ratings from stringent/lenient reviewers and the ‘true’ ratings for several reviewers in experiments.

- In order to evaluate the weighted averaged ratings and further the variance of estimation bias, 12 randomly selected different cases (Denoted as case *tr*(*i*), (*i* = 1…12)) about the distribution of the ‘true’ rating of submissions are taken into account and are listed in Fig 6.
- Meanwhile, 4 randomly selected different cases (Denoted as case *h*(*j*), (*j* = 1…4)) about the distribution of the research area specific *h*-index values of reviewers are considered, which are listed in Fig 7.
  We can observe that in this figure the *h*-index function is non-decreasing. This is due to the fact that Strategy 3 in the CAREER approach, the reviewer with a higher research area specific *h*-index will be assigned later. Then without loss of generality, in this experiment, we can assume that the research area specific *h*-index is non-decreasing as the the sequence number of reviewers is increasing.
- For each of the above cases (*tr*(*i*), *h*(*j*)), we have considered 1000 randomly selected different situations about the distribution of difference $\left(r_{k}^{\left(P_{i}\right)}-{r^{*}}^{\left(P_{i}\right)}\right)$. We randomly select 12 out of 1000 situations as examples and plot them in Fig 8.

**Fig 9:**
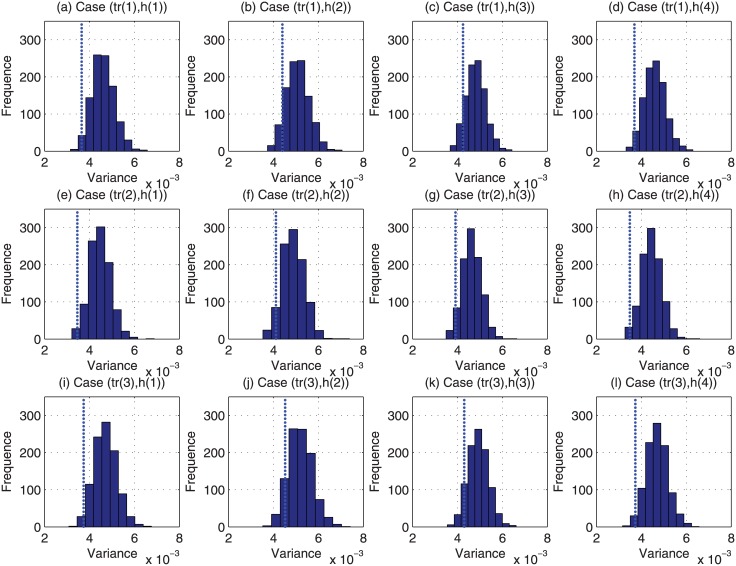
The histograms of the variance of estimation bias for case (*tr*(1), *h*(:)) to case (*tr*(3), *h*(:)) from Monte Carlo based reviewer assignment approach in experiments.

**Fig 10:**
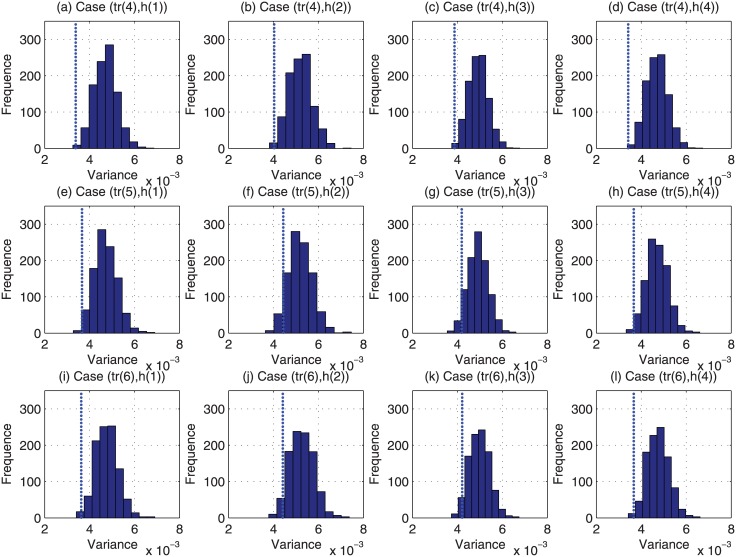
The histograms of the variance of estimation bias for case (*tr*(4), *h*(:)) to case (*tr*(6), *h*(:)) from Monte Carlo based reviewer assignment approach in experiments.

**Fig 11:**
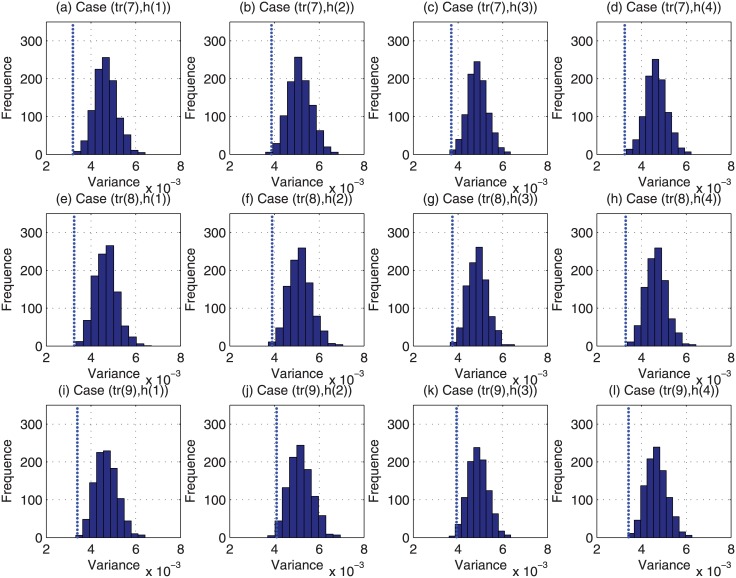
The histograms of the variance of estimation bias for case (*tr*(7), *h*(:)) to case (*tr*(9), *h*(:)) from Monte Carlo based reviewer assignment approach in experiments.

**Fig 12:**
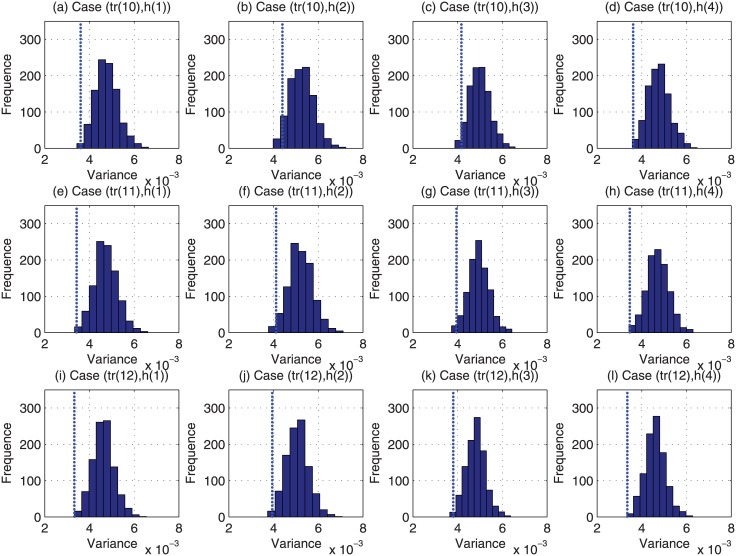
The histograms of the variance of estimation bias for case (*tr*(10), *h*(:)) to case (*tr*(12), *h*(:)) from Monte Carlo based reviewer assignment approach in experiments.

For each of the above 48000 (i.e., 4 × 12 × 1000) cases, we have run the Monte Carlo based reviewer assignment approach 1000 times, and the histograms of their obtained variance of estimation bias are plotted in Figs 9–12. Regarding the CAREER approach, the obtained variance of estimation bias is also plotted as vertical lines in Figs 9–12. Then we list in Table 2 the outperformance percentage of the CAREER approach over the Monte Carlo based reviewer assignment approach for each case.

**Table 2:** The outperformance of CAREER approach in Experiment 1.

	*h*(1)		*h*(2)		*h*(3)		*h*(4)	
	Outperformance percentage	Figure	Outperformance percentage	Figure	Outperformance percentage	Figure	Outperformance percentage	Figure
*tr*(1)	98.30%	Fig 9(a)	91.50%	Fig 9(b)	90.40%	Fig 9(c)	98.30%	Fig 9(d)
*tr*(2)	98.90%	Fig 9(e)	94.70%	Fig 9(f)	96.10%	Fig 9(g)	98.50%	Fig 9(h)
*tr*(3)	98.20%	Fig 9(i)	90.00%	Fig 9(j)	91.50%	Fig 9(k)	98.30%	Fig 9(l)
*tr*(4)	99.90%	Fig 10(a)	99.10%	Fig 10(b)	99.60%	Fig 10(c)	99.90%	Fig 10(d)
*tr*(5)	98.90%	Fig 10(e)	92.80%	Fig 10(f)	95.00%	Fig 10(g)	99.00%	Fig 10(h)
*tr*(6)	99.00%	Fig 10(i)	95.70%	Fig 10(j)	96.90%	Fig 10(k)	99.30%	Fig 10(l)
*tr*(7)	100%	Fig 11(a)	99.40%	Fig 11(b)	99.90%	Fig 11(c)	100%	Fig 11(d)
*tr*(8)	100%	Fig 11(e)	99.70%	Fig 11(f)	99.80%	Fig 11(g)	100%	Fig 11(h)
*tr*(9)	99.80%	Fig 11(i)	98.90%	Fig 11(j)	99.20%	Fig 11(k)	99.80%	Fig 11(l)
*tr*(10)	99.80%	Fig 12(a)	96.20%	Fig 12(b)	97.40%	Fig 12(c)	99.80%	Fig 12(d)
*tr*(11)	99.80%	Fig 12(e)	98.30%	Fig 12(f)	98.80%	Fig 12(g)	99.90%	Fig 12(h)
*tr*(12)	100%	Fig 12(i)	99.40%	Fig 12(j)	99.40%	Fig 12(k)	99.90%	Fig 12(l)

Taking (*tr*(1), *h*(1)) as an example, from Table 2, we can notice that the outperformance percentage is 98.30%, i.e., the result delivered by our proposed reviewer assignment is better than 983000 out of 1000000 results delivered by the Monte Carlo based reviewer assignments.

With the data listed in Table 2 and depicted in Figs 9–12, we can observe that the proposed CAREER approach can lead to a less variance of estimation bias than that in almost all the Monte Carlo based reviewer assignments. More specifically, the variance of estimation bias delivered by our proposed CAREER approach is better than the one delivered by the Monte Carlo based reviewer assignment approach in at least 90.00% executions. Hence, the CAREER approach can greatly reduce the estimation bias caused by stringent reviewers and lenient reviewers, though we assume that lenient reviewers or stringent reviewers cannot be identified explicitly beforehand.

### Experiment 3—Study on CONCERT Approach

In this experiment, taking into account the expertise relevance between reviewers and submissions, we compare our proposed CONCERT approach with the existing reviewer assignment approach [8], to illustrate that our CONCERT approach can reduce the estimation bias caused by stringent reviewers and lenient reviewers, and hence the variance of estimation bias between the ‘true’ ratings of submissions and the ratings provided by reviewers.

In the reviewer assignment approach proposed in [8], both the expertise relevance between reviewers and submissions, and the expertise of reviewers (i.e., the research area specific *h*-index of reviewers) have also been considered.

In this experiment, in order to compare the different approaches, we use the weighted average ${\hat{r}}^{\left(P_{i}\right)}$ of ratings to estimate the ‘true’ rating of a submission *P*_*i*_,

$$\begin{array}{c} {\hat{r}}^{\left(P_{i}\right)}=\frac{\sum_{k=k_{n}}^{m_{max}}\omega_{k}^{\left(P_{i}\right)}\cdot r_{k}^{\left(P_{i}\right)}}{\sum_{k=k_{n}}^{m_{max}}\omega_{k}^{\left(P_{i}\right)}} \end{array}$$ (15)

where the weight is $\omega_{k}^{\left(P_{i}\right)}=h(R_{j})*D(d_{R_{j}}||d_{P_{i}})$. This is because the higher the *h*(*R*_*j*_), the more likely for reviewer *R*_*j*_ to be trusted to give an accurate rating for the reviewed submission; the larger the *D*(*d*_*R*_*j*__∣∣*d*_*P*_*i*__), the better reviewer *R*_*j*_ will understand the quality of submission *P*_*i*_, and the more likely for for him/her to write objective reviews. Hence, the larger $\omega_{k}^{\left(P_{i}\right)}$, the more likely for reviewer *R*_*j*_ to give a rating which is the same as the ‘true’ rating for the reviewed submission. Therefore, both the research area specific *h*-index *h*(*R*_*j*_) of a reviewer *R*_*j*_ and the expertise relevance *D*(*d*_*R*_*j*__∣∣*d*_*P*_*i*__) between reviewer *R*_*j*_ and submission *P*_*i*_ should be taken into account for providing a rating with less estimation bias from the ‘true’ rating of a submission.

In this experiment, we have considered the following situations in experiment settings.

**Fig 13:**
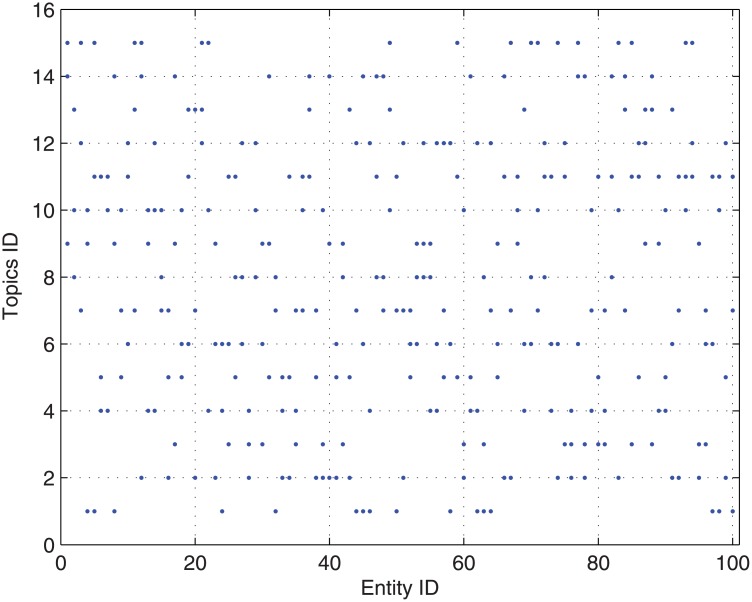
The corresponding topics for each submission in Experiment 2.

**Fig 14:**
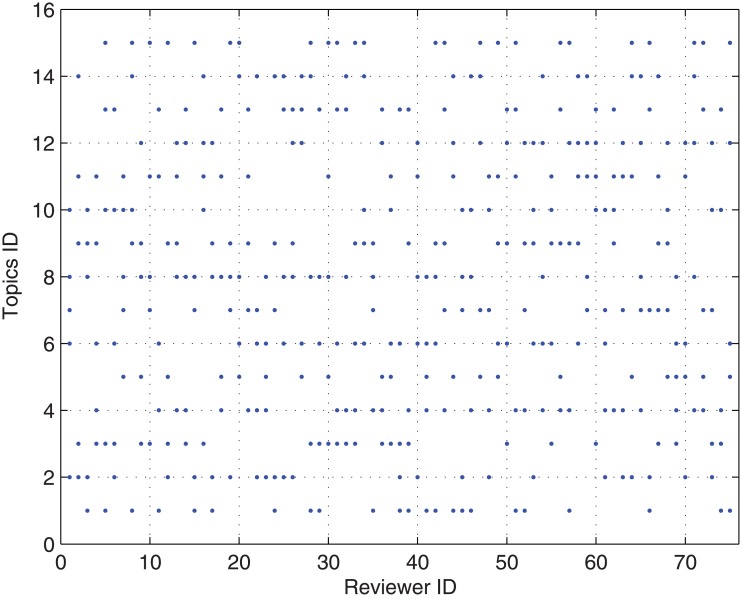
The corresponding topics for each reviewer in Experiment 2.

- We take case *tr*(1) of the ‘true’ rating of submissions and case *h*(1) of the research area specific *h*-index in Experiment 1 as an example to illustrate our CONCERT approach.
- We assume that there are 3 topics for each submission and there are 15 topics in total for all submissions. For each submission, the corresponding topics are depicted in Fig 13.
- We assume that there are 5 topics covered by each reviewer and there are 15 topics in total covered by all reviewers. For each reviewer, the corresponding topics are depicted in Fig 14.

In this experiment, we can obtain that the estimation bias caused by stringent reviewers and lenient reviewers delivered by the reviewer assignment proposed in [8] is 4.5367 × 10^−3^. By contrast, the estimation bias delivered by the CONCERT approach is 3.4376 × 10^−3^, which is 31.97% less. That is due to the fact that in Experiment 2, the expertise relevance between reviewers and submissions has been taken into account. It enhances the effectiveness of cross-assignment, and helps reduce the estimation bias caused by stringent reviewers and lenient reviewers, and thus the variance of estimation bias.

To sum up, we can conclude that the cross-assignment adopted in the CONCERT approach can deliver further improvement compared to the existing reviewer assignment approach [8].

## Conclusions

In the literature, there are many problems in peer review systems, essentially on how to assign the reviewers and aggregate the ratings from both stringent reviewers and lenient reviewers to estimate the ‘true’ rating of a submission, especially when no prior knowledge exists about the distribution of stringent reviewers and lenient reviewers.

In this paper, the academic contexts of reviewers, including research area specific expertise, institution relevance, co-authorship relevance and expertise relevance, are taken into account. In order to reduce the estimation bias caused by stringent reviewers and lenient reviewers, with the CONCERT approach, the ‘true’ rating of a submission can be well estimated, even though no prior knowledge exists about the distribution of stringent reviewers and lenient reviewers.

Experimental results have demonstrated that our proposed CONCERT approach is more reasonable than existing reviewer assignment approaches, and it can greatly help reduce the estimation bias caused by stringent reviewers and lenient reviewers from the ‘true’ rating, i.e., the variance of estimation bias of the ratings provided by these reviewers. This leads to trust enhanced peer selection.

In our future work, the mathematical proof of the effectiveness of cross-assignment will be studied. In addition, effective academic context-aware journal reviewer assignment approaches will also be studied.
